# High-content imaging of presynaptic assembly

**DOI:** 10.3389/fncel.2014.00066

**Published:** 2014-03-03

**Authors:** Vivian Y. Poon, Chiatzun Goh, P. Mathijs Voorhoeve, Marc Fivaz

**Affiliations:** ^1^Neuroscience and Behavioral Disorders Program, Duke-NUS Graduate Medical School, Singapore, Singapore; ^2^Cancer and Stem Cell Biology Program, Duke-NUS Graduate Medical SchoolSingapore, Singapore; ^3^Department of Biochemistry, National University of SingaporeSingapore, Singapore; ^4^Department of Physiology, National University of SingaporeSingapore, Singapore

**Keywords:** synapse formation, presynaptic terminals, image processing, computer-assisted, synaptic vesicles, synaptogenic proteins, microRNA (miRNA), primary neuron culture

## Abstract

Presynaptic assembly involves the specialization of a patch of axonal membrane into a complex structure that supports synaptic vesicle exocytosis and neurotransmitter release. In mammalian neurons, presynaptic assembly is widely studied in a co-culture assay, where a synaptogenic cue expressed at the surface of a heterologous cell induces presynaptic differentiation in a contacting axon. This assay has led to the discovery of numerous synaptogenic proteins, but has not been used to probe neuronal mechanisms regulating presynaptic induction. The identification of regulatory pathways that fine-tune presynaptic assembly is hindered by the lack of adequate tools to quantitatively image this process. Here, we introduce an image-processing algorithm that identifies presynaptic clusters in mammalian co-cultures and extracts a range of synapse-specific parameters. Using this software, we assessed the intrinsic variability of this synaptic induction assay and probed the effect of eight neuronal microRNAs on presynaptic assembly. Our analysis revealed a novel role for miR-27b in augmenting the density of presynaptic clusters. Our software is applicable to a wide range of synaptic induction protocols (including spontaneous synaptogenesis observed in neuron cultures) and is a valuable tool to determine the subtle impact of disease-associated genes on presynaptic assembly.

## Introduction

Chemical synapses are specialized cellular junctions that permit information flow from one neuron to another. They consist of pre- and post-synaptic compartments, whose function is to release and respond to neurotransmitters, respectively. During synaptic transmission, an action potential triggers the entry of calcium ions into the presynapse, which induces synaptic vesicle (SV) fusion and release of neurotransmitters into the synaptic cleft. This complex multi-step process is highly regulated and involves a large number of molecular components. Proteomics studies indicate that hundreds of proteins, comprising scaffolds, receptors, channels, and transporters associate with presynaptic membranes and SVs (Takamori et al., 2006; Morciano et al., 2009; Boyken et al., 2013). Given the diverse array of components present at presynaptic sites, the process of transporting these proteins, stabilizing them at specific sites, and regulating their activity must be tightly orchestrated. Growing evidence suggests that subtle defects in presynaptic function may lead to neurodevelopmental and neurodegenerative diseases. For instance, SV components and molecules required for SV exocytosis, endocytosis, and recycling have been implicated in Alzheimer's disease, schizophrenia, Parkinson's disease, and other disorders (Waites and Garner, 2011).

Genetically tractable model systems like *C. elegans* and *Drosophila* have led to the identification of several evolutionarily conserved cues critical for presynapse formation and function (Chia et al., 2013; Poon et al., 2013). While these forward genetic screens have proved successful in delineating the mechanisms underlying synaptogenesis, they also have several limitations. Firstly, the bidirectional nature of signaling at the synapse makes it difficult to determine whether effects observed are direct and if they are specific to the pre- or post-synapse. Secondly, mechanisms underlying presynaptic assembly in genetically tractable organisms may not always be conserved in the mammalian nervous system.

Synaptogenesis in mammals is extensively studied in dissociated cultures of rodent primary neurons. Although neuron cultures do not retain the physiological organization of brain circuits, they have provided remarkable insight into the molecular mechanisms underlying synaptogenesis. These mechanisms have by and large been confirmed in slice cultures and *in vivo* (Fischer et al., 1998; Dunaevsky et al., 1999; Majewska and Sur, 2003). One approach that has been instrumental in the discovery of synaptogenic adhesion complexes is the use of co-cultures of neurons and heterologous cells (Scheiffele et al., 2000; Biederer et al., 2002; Graf et al., 2004; Kayser et al., 2006; Kim et al., 2006; Linhoff et al., 2009; Kalashnikova et al., 2010). In these mixed cultures, candidate synaptogenic proteins are expressed in heterologous cells and their ability to induce synaptogenesis in contacting neurons is assessed by immunostaining of synaptic markers (Biederer and Scheiffele, 2007). These assays are primarily used as binary read-outs to screen for synaptogenic proteins, and the potential for these assays to provide a quantitative and sensitive measure of synaptogenesis has been largely ignored. One main reason for this is the lack of adequate tools to image this process in a high-content manner, where multiple parameters of presynaptic assembly are extracted for large populations of hemi-synapses. None of the commercially available softwares including Image J and Metamorph have built-in algorithms to detect synaptic assembly in co-culture assays. Hence, synaptogenesis is usually assessed manually, or semi-automatically, in small sample sizes, precluding the analysis of subtle phenotypes. Combining synaptic induction assays with high-content imaging could potentially unravel cue-dependent mechanisms of synapse formation and should in principle allow detection of subtle effects of disease-associated genes on presynaptic assembly.

One class of molecules reported to have a subtle, yet significant impact on synaptic function are microRNAs (miRNAs) (Schratt, 2009). These short non-coding RNAs are highly expressed in the brain (Chiang et al., 2010) and regulate the majority of coding transcripts (Friedman et al., 2009). Each miRNA is predicted to target hundreds of transcripts (Lim et al., 2005; Rajewsky, 2006), and target genes typically have putative binding sites for several different miRNAs (Tsang et al., 2010). miRNA networks are thus highly distributed, implying that each individual miRNA typically has a mild impact on its target genes. Nevertheless, miRNAs have been implicated in neuronal and synaptic development (Fineberg et al., 2009) and are associated with several brain disorders (Im and Kenny, 2012). Whether miRNAs participate in the assembly of a presynaptic terminal is yet to be explored.

Here, we introduce an image-processing algorithm that reliably detects synaptic clusters in co-culture assays and extracts several presynaptic parameters. We used this software to examine the impact of eight neuronal miRNAs on presynaptic induction and identified a novel role for miR-27b in elevating the density of cue-induced presynaptic clusters. Finally, we demonstrate the versatility of our script by quantifying spontaneous synaptogenesis in mature primary neurons.

## Results

### An image-processing software to detect presynaptic clusters in co-culture assays

**Figure 1:**
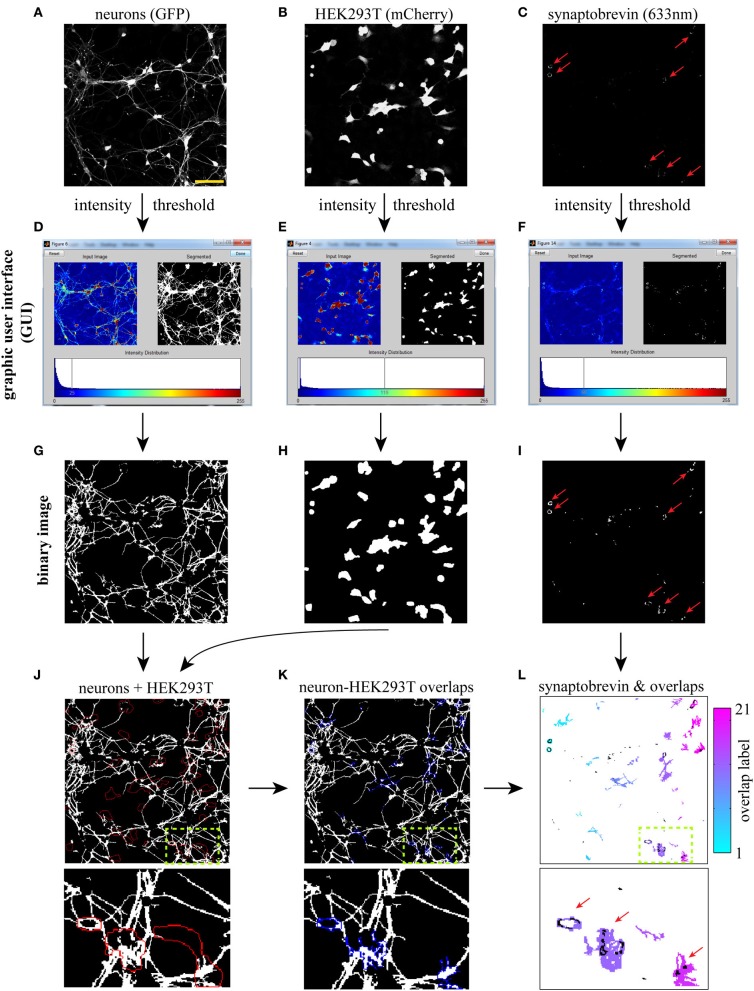
**Workflow for computer-assisted segmentation of presynaptic clusters**. The three input images (20×) **(A–C)** are binarized using an interactive graphic user interface (GUI, **D–F**), and the resulting binary images are cleaned-up (see text) using morphological openings procedures **(G–I)**. **(J)** Overlay of neuron with red outline of HEK293T cells. **(K)** Neuron-HEK293T overlap regions outlined in blue. **(L)** Individual overlap regions are pseudocolored (the color map refers to the number of regions detected in the field) and overlaid with binarized synaptic vesicles (SVs, in black). A small region of the 20× field (dotted box) is blown up to facilitate visualization of individual overlap regions and associated SVs. The software then extracts several SV cluster features for each overlap area, such as size, intensity and density. It also calculates the fraction of overlap regions containing one or more SV clusters. Synaptobrevin puncta detected outside overlap regions (and thus not induced by LRRTM2) are not included in the analysis. Red arrows point to LRRTM2-induced SV clusters. Scale bar, 50 μm.

To induce presynapse formation in primary neurons, we adapted the co-culture protocol first described by Biederer and Scheiffele (2007). Hippocampal neurons were cultured with an astroglial feeder layer to accelerate neuronal differentiation (Kaech and Banker, 2006) and transduced with a GFP-expressing lentivirus to visualize neuronal processes (Figure 1). HEK293T cells, co-electroporated with a synaptogenic cue and mCherry, were added to neuron cultures after 6 days *in vitro* (DIV). We chose the leucine rich repeat transmembrane protein 2 (LRRTM2) as the post-synaptic cue, as it was previously demonstrated to induce robust differentiation of functional presynaptic terminals (De Wit et al., 2009; Ko et al., 2009; Siddiqui et al., 2010). Neurons were fixed at DIV8, stained with an antibody against the synaptic vesicle (SV) protein synaptobrevin and imaged by confocal microscopy (Supplemental Figure 1).

To detect hemi-synapses induced by LRRTM2, we wrote an image-processing algorithm in Matlab, which uses information from three input images: neurons (GFP), HEK293T cells (mCherry) and synaptobrevin (633 nm). These images can be acquired at high magnification (63×, 100×), or at a lower power (down to 20×), depending on the application; high magnification resolves smaller SV clusters, while lower power yields larger fields and increases sample size.

We opted for a lower power (20×) to illustrate the workflow of our image segmentation approach. Our algorithm currently reads 3-color RGB stacks containing the three input images in lsm (Zeiss) or tif formats (Figures 1A–C). The first step consists of binarizing these three input images. A global intensity threshold is automatically computed for each image and is easily modifiable through an interactive graphic user interface (GUI) (Figures 1D–F). This GUI allows users to bring out relevant cellular features by moving the cursor along the intensity distribution scale. Typically, a low threshold value is chosen for the GFP image, in order to bring out relatively dim neuronal processes (Figures 1A,D). A higher threshold value is used, in contrast, for the synaptobrevin images to specifically segment SV clusters (Figures 1C,F). These threshold values will depend on a variety of factors, such as GFP or mCherry expression, background in SV antibody staining, magnification or imaging system, and thus have to be determined by the user for each independent experiment. Once a set of thresholds has been chosen, we recommend the use of fixed thresholds across all images within one experiment, to exclude threshold-dependent variation in segmentation outputs (Supplemental Figure 2) and support automated workflow during batch-processing analysis of multiple images. We provide to this end a version of the script which operates with fixed, user-defined threshold values (Supplemental data 1).

Three binary images are then generated based on user-defined thresholds (Figures 1G–I) and “cleaned up” using the following procedures (see Materials and Methods). All three binary images are subjected to a “morphological opening” (an erosion followed by a dilation) in order to eliminate objects below a certain size. To restrict analysis to neuronal processes, cell bodies are specifically eliminated from the GFP binary image using a morphological opening approach (Figure 1G). The HEK293T binary image is then slightly dilated (Figure 1H) to facilitate detection of SV clusters that are formed along the edge of HEK293T cells.

The second step involves the generation of overlap regions between neuronal processes (i.e., axons) and HEK293T cells. These overlap regions are computed by applying the logical “AND” operator to the HEK293T (mCherry) and neuron (GFP) binary images (Figures 1J,K). A small region of the 20× field is blown up to illustrate this process. These overlap regions are gated by size (small overlaps are discarded) and labeled as individual objects, which allows one to derive SV cluster data for each individual overlap region (Figure 1L). Several parameters can then be extracted for each of the detected overlap regions, including SV cluster size, intensity, and density (fraction of overlap area covered with SVs). These data are then automatically exported to an excel spreadsheet, which lists all labeled areas with their corresponding SV features.

We chose to focus on two relevant measures of presynaptic assembly—the fraction of overlap regions containing one or more SV clusters (frequency) and the fractional area of overlap regions occupied by SV clusters (density). Both the frequency and density of SV clustering reflect the probability of an axonal segment to differentiate into a presynaptic terminal when in contact with a given cue. SV cluster density also depends on the size of induced SV clusters. Because the size of a presynaptic terminal correlates with its synaptic vesicle and quantal content (Petrof and Sherman, 2013), SV cluster density is also indicative of presynaptic maturation.

### Evaluating the performance of our SV cluster detection algorithm

**Figure 2:**
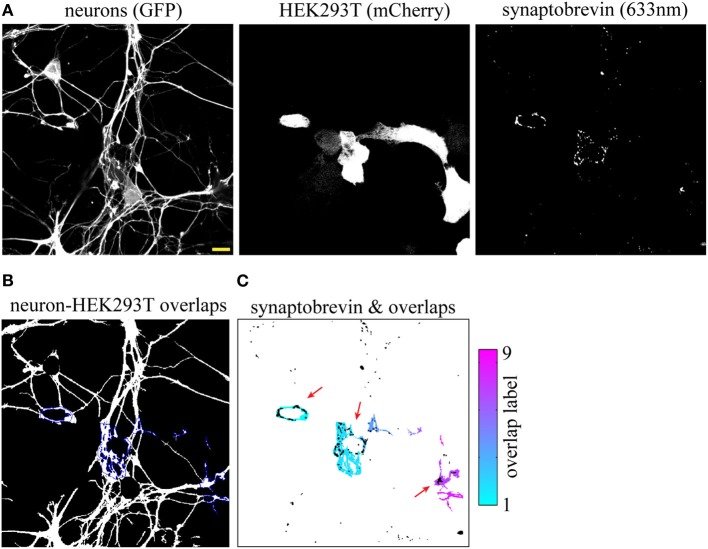
**Synaptic vesicle (SV) cluster detection using high (63×) magnification images. (A)** Input images. The field shown corresponds to the one magnified in Figures 1J–L. **(B)** Neuron-HEK293T overlap regions outlined in blue. **(C)** Individual overlap regions are pseudocolored and overlaid with SV clusters (black). Red arrows point to LRRTM2-induced SV clusters. Scale bar, 5 μm.

To compare segmentation of SV clusters at 20× and 63×, we imaged the same field at higher magnification and fed these three 63× images (Figure 2A) to our SV cluster detection algorithm. The field shown in Figures 2A–C corresponds to the one blown up in Figures 1J–L. Note that the three main overlap regions and their corresponding SV clusters (indicated by red arrows in Figures 1L, 2C) are equally well-detected at 20× and 63×. However, the increased resolution from the higher magnification results in a finer map of overlap regions and associated SVs. Given the numerical aperture (NA) of our objectives and the resolution of our detector (i.e., pixel size) SV clusters down to ~300 n are detectable at 63×, whereas segmentation at 20× resolves clusters of ~1 μm and larger.

To evaluate the performance of our script on 20 and 63× images, we compared manual and computer-assisted detection of SV clusters. ROIs (regions of interest) for neuron-HEK293T overlap regions and SV clusters were drawn (manually) in Metamorph and relevant parameters were exported to an excel spreadsheet to calculate the average SV cluster frequency and density. The same fields were then fed to our algorithm which automatically extracted these two parameters. The script measured SV cluster frequency and density with an accuracy of 87% or higher for both magnifications (Table 1). The accuracy and sensitivity of this algorithm depends on the threshold values used for segmentation (Figures 1D–F and Supplemental Figure 2). High threshold values (stringent segmentation) exclude dim overlap regions and associated SV clusters. Low threshold values, in contrast, (permissive segmentation) may lead to artifactual detection of overlap regions and SVs. We therefore recommend users to run test trials on a small data set to identify adequate thresholds before analyzing large data sets with this script.

**Table 1:** **Comparison of SV cluster frequency and density obtained using manual vs. computer-assisted processing of low (20×) or high (63×) magnification images (number of overlap regions = 40–140)**.

**SV cluster**	**Magnification**	**Manual**	**Computer-assisted**	**Accuracy (%)**
Frequency	20×	0.61	0.62	97
Density	20×	0.18	0.20	91
Frequency	63×	0.75	0.68	90
Density	63×	0.16	0.14	87

This side-by-side comparison allowed us to estimate the time gained by automating the analysis of SV cluster frequency and density. Manual segmentation of ~200 overlap regions (and associated SVs) on 20× images takes roughly 3 h. The script analyses these images in 30 s or faster (depending on processor speed), roughly 360 times faster.

### Evaluating the variability of presynaptic induction using the SV clusters detection algorithm

**Figure 3:**
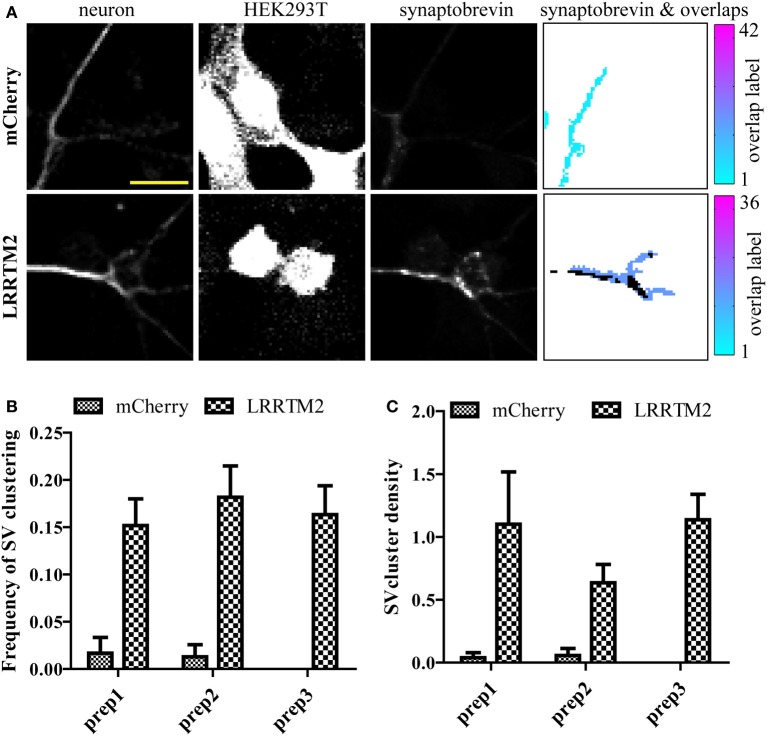
**Evaluating the variability of presynaptic induction using software-based analysis of synaptic vesicle (SV) clusters induced by LRRTM2. (A)** Raw confocal images (20×) of GFP-expressing neurons cultured with HEK293T cells transfected with mCherry alone (top row) or mCherry and LRRTM2 (bottom row) and immunostained against synaptobrevin. The overlap region map and corresponding SV clusters (black) is displayed on the right. Scale bar, 20 μm. **(B)** Mean percentage of axon-HEK293T overlap regions containing SV clusters (SV cluster frequency) from six different fields, across three independent experiments. HEK293T cells express mCherry (control) or mCherry together with LRRTM2. *p* < 0.001, ANOVA. No significant difference (*p* = 0.78) between the three independent neuronal preparations. **(C)** Fraction of area of overlap region occupied by SV clusters (SV cluster density). *p* < 0.05, ANOVA. No significant difference (*p* = 0.44) between the three independent neuronal preparations.

While LRRTM2 robustly induces presynaptic assembly, it is unclear if the inherent variability of this assay may hinder its use to study presynaptic assembly. To address this issue, we quantified the frequency and density of SV clustering in neurons co-cultured with HEK293T cells expressing either a control mCherry plasmid or mCherry together with LRRTM2, in three independent neuronal preparations. We imaged six 20× fields for each condition and processed the images with our SV clusters detection algorithm. We observed a clear induction of presynaptic assembly, as measured by a significant increase in both the frequency and density of SV clustering in the presence of the synaptogenic cue (Figures 3A–C). Average values for frequency and density were remarkably similar in these three different neuronal preparations. Together, these results indicate that the analysis of a sufficient number of fields (5–10 fields per condition) leads to accurate and reproducible quantification of presynaptic induction, suggesting that this synaptic induction assay combined with automated detection of presynaptic clusters is capable of detecting subtle alterations in synapse formation.

### Software-based detection of SV clusters reveals subtle effects of miRNAs on presynaptic assembly

We next used this software to probe the impact of miRNAs on presynaptic assembly. As a pilot screen, we selected eight miRNAs that are expressed in mammalian neurons and predicted to target disease-associated presynaptic genes (Kim et al., 2004; Miska et al., 2004; Dogini et al., 2008; Manakov et al., 2009; Chiang et al., 2010)—miR-145, 195, 196a, 196b, 218, 27b, 324, and 92a. The data mining approach employed to identify these miRNAs is further described in the Materials and Methods section. To overexpress these miRNAs, we generated lentiviral miRNA expression constructs, which co-express GFP to allow visualization of transduced neurons. We obtained high-efficiency transduction of neurons with these lentiviruses, which had no detectable impact on cell health, morphology and number.

Three miRNA constructs, miR-196a, miR-27b, and miR-324, were randomly chosen to confirm overexpression of miRNAs in neurons. Two methods were used- quantitative PCR (qPCR) and luciferase assays. Using qPCR, we observed increases in the levels of mature miR-196a-5p, miR-27b-5p, and miR-324-5p in neurons transduced with lentiviruses specific for these miRNAs (Supplemental Figure 3A). In addition, we generated luciferase sensors containing sequences complementary to the miRNA and luciferase activity was measured in primary neurons. Overexpression of miR-196a leads to down-regulation of luciferase activity in both the miR-196a-5p and miR-196a-3p sensors, indicating the production of mature miRNAs from the exogenously expressed miRNA construct that bind to the sensor and reduce luciferase expression (Supplemental Figure 3B).

**Figure 4:**
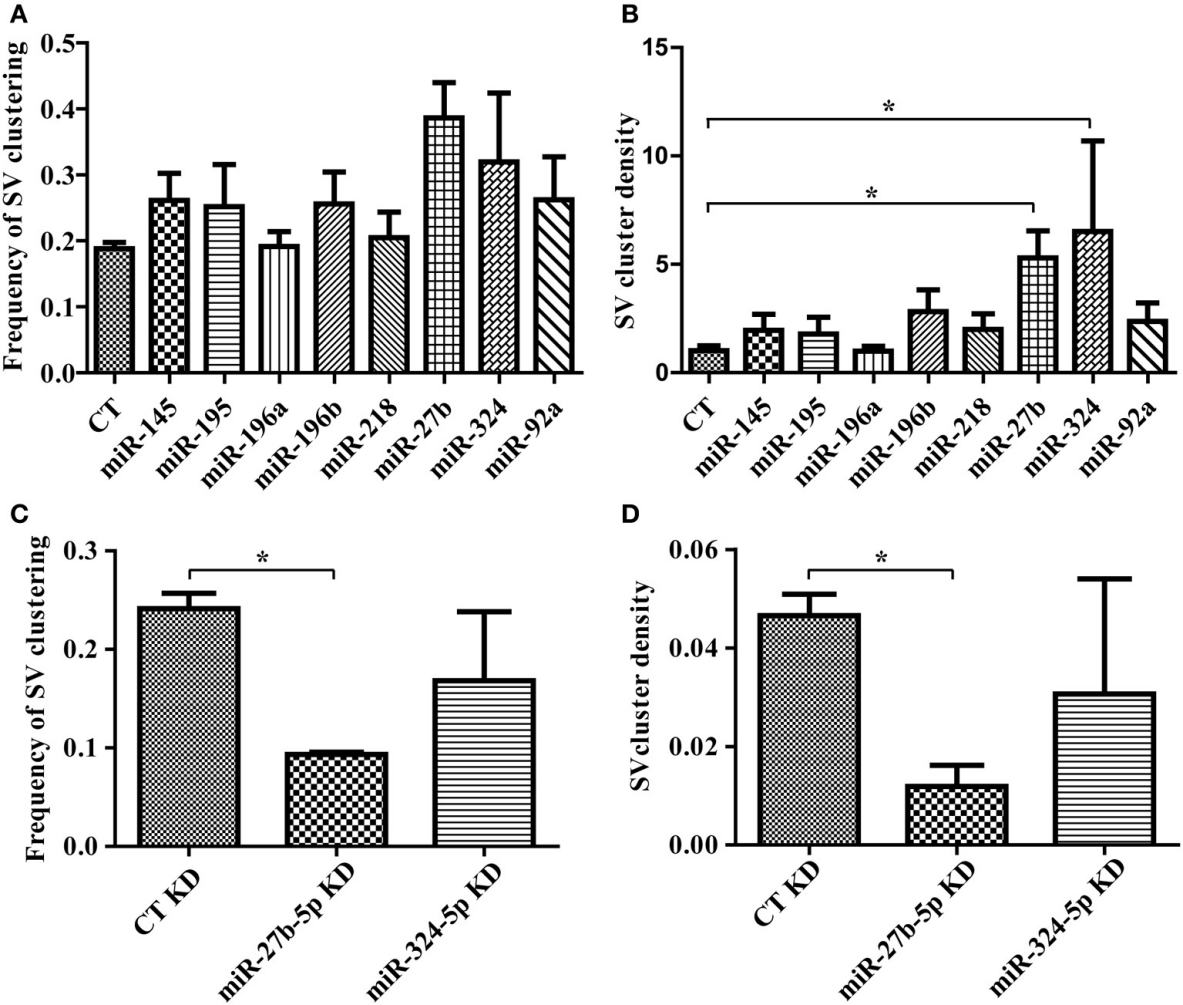
**Software-based detection of synaptic vesicle (SV) clusters reveals specific effects of miRNAs on presynaptic assembly. (A,B)** Hippocampal neurons were transduced with viruses expressing the empty vector (CT) or miRNAs, cultured with HEK293T cells transfected with mCherry alone or mCherry with LRRTM2 and immunostained against synaptobrevin. *n* = 3–6 independent experiments for each condition and six fields (20×) were imaged. **(A)** Frequency of SV clusters in the presence of LRRTM2. *p* > 0.05, ANOVA. **(B)** Mean SV cluster density. *p* = 0.0212, ANOVA. ^*^p < 0.05, *t*-test. **(C,D)** Hippocampal neurons were transduced with GFP virus, transfected with miRNA inhibitors, cultured with HEK293T cells transfected with mCherry alone or mCherry with LRRTM2 and immunostained against synaptobrevin. *n* = 2 independent experiments for each condition and six fields (20×) were imaged. Frequency **(C)** and density **(D)** of SV clustering is shown. ^*^p < 0.05, *t*-test.

Having confirmed that our lentiviral vector leads to robust expression of miRNAs in neurons, we next evaluated the impact of our miRNA set on synaptic induction. We transduced miR-145, 195, 196a, 196b, 218, 27b, 324, or 92a in primary hippocampal neurons, added HEK293T cells expressing mCherry alone, or mCherry with LRRTM2, and measured the frequency and density of SV clusters. LRRTM2-induced increase in frequency of SV clustering was not affected by any of these miRNAs (Figure 4A). Overexpression of miR-27b and miR-324 led to higher frequency of SV clustering but this increase, when compared with control neurons, is not statistically significant. While the frequency of SV clusters is largely unaltered, the density of SV clusters is significantly increased in neurons overexpressing miR-27b or miR-324 (Figure 4B). To further investigate the role of miR-27b and miR-324 on presynaptic induction, we obtained hairpin inhibitors specific for miR-27b-5p and miR-324-5p and verified their ability to knock down their target miRNA (Supplemental Figure 3C). We next transfected these inhibitors into primary neurons, added HEK293T cells expressing mCherry alone, or mCherry with LRRTM2, and measured the frequency and density of SV clusters. Knockdown of miR-27b-5p decreases frequency and density of SV clusters while knockdown of miR-324-5p has no significant effect (Figures 4C,D). Though the miR-324-5p inhibitor leads to loss of approximately 70% of miR-324-5p (Supplemental Figure 3C), absolute levels of this miRNA remain high after knockdown (due to high endogeneous expression, Supplemental Figure 3A) and could still be sufficient to promote presynaptic induction. Taken together, we identified a novel role for miR-27b in promoting presynaptic assembly through gain- and loss-of-function approaches, combined with our SV clusters detection algorithm.

### Detection of endogenous synapses in mature neuron cultures

**Figure 5:**
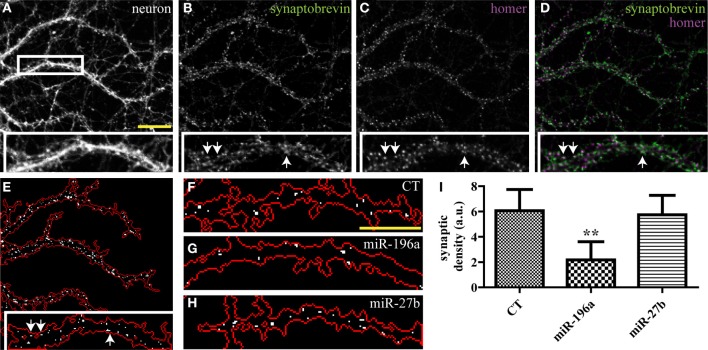
**Quantifying the impact of miRNAs on endogenous synapses in mature hippocampal neurons**. Hippocampal neurons were transduced with viruses expressing the empty vector (CT), miR-196a, or miR-27b, and immunostained (DIV21) against pre- and post-synaptic markers. **(A–C)** Raw confocal images (63×) of DIV21 hippocampal neurons. Scale bar, 5 μm. Inset, magnified view of boxed area. **(D)** Merged image of SV protein synaptobrevin (green) and post-synaptic protein homer (purple). **(E)** Output image of our synapse detection algorithm showing the neuronal outline (red) and synapses (white puncta). **(F–H)** Representative images of synapses detected in dendrites of neurons transduced with an empty vector **(F)**, miR-196a **(G)**, or miR-27b **(H)**. Scale bar, 5 μm. **(I)** Quantification of synaptic density. *n* = 3. ^**^p < 0.01, ANOVA.

While the SV clusters detection program was initially developed for the purpose of quantifying presynaptic induction in co-cultures, it can be easily modified to detect synapses in other preparations. Here, we use a variant of this script to segment excitatory synapses in mature hippocampal neurons, based on overlap between pre- and post-synaptic markers. This modified script requires three high-resolution (60× or greater) input images consisting of a neuronal (GFP), pre-synaptic (synaptobrevin) and post-synaptic (homer) markers (Figures 5A–D and Supplemental data 1). The program displays a map of detected synapses (Figure 5E) and computes the average synaptic density. We then used this program to quantify the impact of miRNAs on synaptogenesis and found that overexpression of miR-196a led to a marked reduction of synaptic density, while miR-27b had no detectable effects (Figures 5F–I and Supplemental Figure 4).

Though miR-27b affects presynaptic induction in young neurons, it has no effect on the presynapse in mature neurons. It is possible that miR-27b only functions transiently during development in promoting presynaptic assembly, and that accelerated presynaptic development does not result in a detectable modification of synaptic density in mature neurons. Alternatively, it is also plausible that miR-27b is specifically involved in LRRTM2-mediated presynaptic induction and that other endogenous synaptogenic cues mask the impact of miR-27b on synaptic assembly. Finally, a stronger overexpression of miR-27b may be required to observe effects on endogenous mature synapses.

Collectively, use of these synapse detection algorithms allowed the identification of distinct roles for miR-27b and miR-196a in cue-induced presynaptic induction and spontaneous synaptogenesis, respectively.

## Discussion

Imaging approaches are widely used to study synaptogenesis in different organisms and neuronal preparations. The local nature of synaptic assembly and the heterogeneity in morphological outcomes (synapse size, shape, and time of differentiation) render this process difficult to capture by biochemical or other bulk-population techniques. Imaging gives, in principle, access to information at the single-synapse level in large populations. Yet, a limited number of tools are currently available to automate synapse detection, and data in the literature is by and large analyzed manually or semi-automatically. Much progress has been made in recent years in automated image analysis of tissue-cultured cells (Wong et al., 2010; Evensen et al., 2013; Mokhtari et al., 2013). Computer-assisted analysis of neuronal features lags behind, however, owing to the inherent complexity of neuronal preparations.

We have developed a versatile and robust synapse detection algorithm, which uses simple image segmentation strategies to extract synaptic features in complex neuronal preparations. We describe the use of this software to quantify presynaptic assembly in co-culture assays. Our SV clusters detection script selectively and accurately identifies hemi-synapses that are induced by a synaptogenic cue expressed at the surface of a heterologous cell. Two recent papers describe the use of micropatterned substrates (Czondor et al., 2013) or specialized arrays of cells expressing synaptogenic adhesion molecules (Shi et al., 2011) to improve the sensitivity of this assay. Although our SV clusters detection script requires no specialized arrays or patterned substrates to reliably detect presynaptic induction, it can easily be combined with these innovative approaches.

To explore the possibility of using high-content imaging to identify novel regulators of synaptogenesis, we analyzed the impact of a small set of miRNAs on cue-induced presynaptic assembly and spontaneous synapse formation. We identified two miRNAs- miR-27b and miR-196a, with distinct functions in synaptic development. miR-27b augments SV cluster density, while miR-196a substantially reduces synaptic density in mature hippocampal cultures. While several miRNAs have been implicated in post-synaptic structure and function (Fineberg et al., 2009; McNeill and Van Vactor, 2012), less is known about miRNA function in presynaptic terminals. Our findings, identifying a role of miR-27b in presynaptic induction, suggests that miRNAs may exert important regulatory functions at the presynapse, and are in line with a recent bioinformatics study showing that presynaptic transcripts have unusually long 3′UTRs and an increased number of predicted miRNA target sites (Paschou et al., 2013).

In conclusion, our image-processing software offers a flexible solution for high-content imaging of synapses in various neuronal preparations. Its sensitivity and accuracy enables detection of modest synaptic phenotypes, a property that might be particularly useful to probe the effect of disease-associated genes on synaptic function.

## Materials and methods

### Constructs and generation of lentiviruses

pBOS-myc-hLRRTM2 was a generous gift from A. Ghosh (UCSD) and mCherry-C1 was generated by replacing EGFP in pEGFP-C1 vector (Clontech) with mCherry using the BsrGI and AgeI sites. To overexpress miRNAs, genomic segments encompassing the miRNA of interest and ~200 kb of flanking region were PCR amplified from genomic rat DNA and cloned downstream of GFP and its stop codon in the FUGW GFP lentiviral vector (#14883 from Addgene; Lois et al., 2002) using the EcoRI site. Sequences of the oligos used to generate the miRNA minigenes are listed in Table 2.

**Table 2:** **Oligos for miRNA overexpression and miRNA sensors**.

**Primer**	**Sequence (5′ to 3′)**
pVP109 miR-145 EcoRI F	gaaagggaattcACTGGTCCCAAATGCTTCCTGAC
pVP6 miR-145 EcoRI R	gaaagggaattcAGCAGTTCTGAGGTTCCCACATC
pVP124 miR-195 EcoRI F	gaaagggaattcCACCATCATCATCATCATCTACGG
pVP125 miR-195 EcoRI R	gaaagggaattcTCTAAGCAAGGACTGCACGAG
pVP114 miR-196a EcoRI F	gaaagggaattcGCTAGAGGTTCCATTTCACCAG
pVP115 miR-196a EcoRI R	gaaagggaattcCCTTGGAATTGGTTGGACCTTC
pVP116 miR-196b EcoRI F	gaaagggaattcTTTCACTCTCTCCTCCTAAGCG
pVP117 miR-196b EcoRI R	gaaagggaattcGTCCAAGAATCAGCCGCTAAC
pVP112 miR-218 EcoRI F	gaaagggaattcGCTGCAGTTCTGAGGAACATG
pVP113 miR-218 EcoRI R	gaaagggaattcTGTAAAGTGGGGCTTTCAAGG
pVP118 miR-27b EcoRI F	gaaagggaattcTCCTGGCATGCTGATTTGTGAC
pVP119 miR-27b EcoRI R	gaaagggaattcACTCCGCTCAGCTTTTGTGATG
pVP110 miR-324 EcoRI F	gaaagggaattcTACTCAAGCCTTCAAGACCAGC
pVP20 miR-324 EcoRI R	gaaagggaattcGTCAGCATAGTTAGGTCTCCAG
pVP111 miR-92a-19b-20b EcoRI F	gaaagggaattcCTAGTGCTGTTAGTGAAGCAGC
pVP14 miR-92a-19b-20b EcoI R	gaaagggaattcCATCATAGCCATGACTAACGG
pVP148 miR-196a-5p sensor F	TCGAG cccaacaacatgaaactaccta GC
pVP149 miR-196a-5p sensor R	GGCCGC taggtagtttcatgttgttggg C
pVP150 miR-196a-3p sensor F	TCGAG tcaggcagtttcttgttgccga GC
pVP151 miR-196a-3p sensor R	GGCCGC tcggcaacaagaaactgcctga C

FUGW lentiviral particles were produced and purified as previously described (Tiscornia et al., 2006). FUGW GFP virus was used as a control (CT). All viral preparations were titrated and multiplicity of infection (MOI) between two and five were used for all viral transduction experiments.

The miR-196a-5p and miR-196a-3p luciferase sensors were generated by ligation of annealed oligos via the XhoI-NotI site in the miR-Sens vector (Beillard et al., 2012). Primers used for cloning are listed in Table 2. All constructs were sequenced before use.

### Data mining approach to isolate miRNAs of interest

A bioinformatic screen was employed to identify miRNAs that are predicted to target disease-associated presynaptic genes and are expressed developmentally in mammalian central nervous system (CNS) neurons. Our final list of miRNAs met the following criteria:

1. Reported to be expressed in developing mammalian CNS neurons by at least three articles (Kim et al., 2004; Miska et al., 2004; Dogini et al., 2008; Manakov et al., 2009; Chiang et al., 2010).
2. Predicted to target disease-associated presynaptic genes by at least three miRNA databases. Disease-associated presynaptic genes were chosen based on a paper by Waites and Garner (2011). Seven prediction programs (DIANAmT, miRanda, miRDB, miRWalk, RNAhybrid, PICTAR4, RNA22, targetscan) were used to identify putative miRNA sites.
3. No previously reported (published) role in neuronal development.

### Cell culture

Dissociated hippocampal and cortical neurons from embryonic E18 rat embryos and primary astrocytes from P0 rat pups were prepared as described (Fivaz and Meyer, 2005; Kaech and Banker, 2006). 30,000 neurons were seeded on each 12 mm-wide poly-L-lysine-coated glass coverslip. Neurons were transduced with lentiviruses 3 h after plating to express cytoplasmic GFP or miRNAs of interest and cultured in glia-conditioned media. For miRNA knockdown experiments, 100 μmoles of miRIDIAN hairpin inhibitors (ThermoScientific) were introduced into neurons by nucleofection using the Rat Neuron Nucleofector kit II (Amaza Biosystems, Lonza, O-003). 125,000 electroporated neurons were seeded on each glass coverslip. Neurons were next transduced with lentiviruses 3 h after plating to overexpress cytoplasmic GFP and cultured in glia-conditioned media.

Coverslips with neurons were transferred to an astroglial feeder layer 1 day later. After 6 days, 400,000 HEK293T cells were transfected using the Neon electroporation system (Life Technologies) with three pulses at 1245 V for 10 ms (300 ng mCherry alone or together with 1.25 μg LRRTM2 per million HEK293T cells). Coverslips with neurons were transferred to separate wells containing glia-conditioned media and the transfected HEK293T cells were added. Once the HEK293T cells attached (a few hours later), coverslips containing neurons and HEK293T cells were returned to an astroglial feeder layer. Co-cultures were processed and analyzed 2 days later.

For the study of endogenous synapses, dissociated hippocampal neurons from E18 rat embryos were seeded on glass coverslips and fixed after 3 weeks in culture.

### Immunocytochemistry

Co-cultures grown on glass coverslips were fixed in 4% paraformaldehyde and 4% sucrose in phosphate-buffered saline solution (PBS) for 20 min. Blocking and permeabilization was done with 5% goat serum and 0.25% TritonX-100. To visualize endogenous SV clusters, cultures were stained with mouse anti-synaptobrevin (Covance MMS-616R clone SP10; 1:10,000) followed by goat Alexa Fluor 633 anti-mouse IgG (Invitrogen, A21050; 1:500). Primary and secondary antibodies were incubated at room temperature for an hour each and cells were mounted on a glass slide for imaging. DIV21 hippocampal neurons were also stained with rabbit anti-homer (Synaptic Systems, 160003; 1:500) followed by goat Alexa Fluor 568 anti-mouse IgG (Invitrogen, A11004; 1:1000).

### Image acquisition

Samples were visualized on an upright laser scanning confocal microscope (LSM710, Zeiss) with a Plan-Apochromat 20× (*NA* = 0.8) objective or a Plan-Apochromat 63× (*NA* = 1.40) oil objective and appropriate filter sets for GFP (488 nm laser, detection bandwidth 500–575 nm), mCherry (543 nm laser, detection bandwidth 570–625 nm) and Alexa633 (633 nm laser, detection bandwidth 650–750 nm). All samples from each neuronal preparation were stained simultaneously and imaged with identical settings. Six random fields were imaged for each sample. To image endogenous synapses in DIV21 cultures, we used a 63× objective and imaged 10 random fields for each sample.

### Image processing

All scripts described in this work are written in Matlab and listed in Table 3. The primary codes are available in Supplemental data 1. These algorithms require the Matlab image processing toolbox and use a few sub-routines that have been written by others and are referenced in Table 3.

**Table 3:** **List of. m files**.

	**Function**	**References**
SVclusters_detection.m	Identifies SV clusters in neuron-HEK293T overlap regions (three color input)	This work
SVclusters_detection_thresholds.m	Similar to above but includes interactive graphic user interface (GUI) to define thresholds	This work
synapse_detection.m	Identifies overlap regions between pre- and post-synaptic markers within neuronal process (three color input)	This work
Thresh_tool	Launches a GUI for thresholding an intensity in an input image	http://www.mathworks.com/matlabcentral/fileexchange/6770-thresholding-tool
bwperim.m	Finds perimeter of objects in binary image	http://www.mathworks.com/access/helpdesk/help/toolbox/images/bwperim.html
imoverlay.m	Overlays a binary mask onto an image using a specified color	http://www.mathworks.com/matlabcentral/fileexchange/10502
vislabel.m	Assigns a number to detected objects	http://www.mathworks.com/matlabcentral/fileexchange/19665-visualize-output-of-bwlabel

#### Intensity thresholding

Thresholding of the three input images is done through a GUI (Thresh_tool) which automatically computes a global threshold level, using the Matlab built-in function graythresh. A line on the histogram points to the current threshold level. Level can be changed by dragging the line left and right. The output image updates automatically. Fixed thresholds can also be specified beforehand, thereby bypassing the interactive GUI.

#### Binary image “clean-up”

All three binary images (GFP, mCherry, and SVs) are subjected to a morphological opening using the bwareaopen function from Matlab, to eliminate objects below a certain size. The default size thresholds should function for most applications but can be further adjusted by users. The neuron (GFP) image consists of processes (axons mostly) and cell bodies. Because we are interested in segmenting axons only, we eliminated cell bodies by performing a morphological opening using a defined structural element, consisting of a disk the size and shape of a cell body. This allows specific segmentation of cell bodies, which are then subtracted from the original GFP binary image to yield a binary image consisting exclusively of neuronal processes. Finally, the HEK293T binary image is slightly dilated, to include detection of SVs that form along the edge of these cells.

#### Generation and display of overlap regions

Neuron-HEK293T overlap regions are computed by performing a logical “AND” operation between the GFP and mCherry image, resulting in a specific mask for overlap regions. Each of these overlap regions is then labeled using the bwlabel function from Matlab and displayed according to a color map (cool) to visualize individual (labeled) regions. The final image output is the map of overlap regions overlaid with the binary SVs shown in black.

#### Data analysis, export and display

Three SV-specific parameters are measured for each overlap area: SV cluster intensity, size and density. These data are then automatically exported to an excel spreadsheet, which lists all labeled areas with their corresponding SV features. Results were then graphically displayed using GraphPad Prism.

### Luciferase assays

These assays were conducted using the Dual-Luciferase Reporter kit (Promega) as described previously (Beillard et al., 2012). Five micrograms of miR-Sens-196a-5p or miR-Sens-196a-3p were introduced into five million primary cortical neurons using nucleofection (Amaxa O-003, Lonza) and one million cells were seeded per well in a poly-L-lysine-coated 24-well cell culture plate. After 5 or 6 days of culture, cells were washed twice with PBS and lysed for 30 min at room temperature in 120 μl of 1× PLB buffer. Fifty microliters of each reagent was added to 10 μl of the lysate in a black 96-well plate (NUNC) and luciferase activity was measured using the Tecan Infinite M200 microplate reader.

### RNA extraction and reverse transcription(RT)-qPCR

Dissociated hippocampal and cortical neurons from embryonic E18 rat embryos were prepared as described (Fivaz and Meyer, 2005; Kaech and Banker, 2006). 500,000 neurons were seeded per well in a poly-L-lysine-coated 12-well cell culture plate and transduced with lentiviruses to overexpress gfp, miR-196a, miR-27b, or miR-324 3 h after plating or electroporated with miRIDIAN hairpin inhibitors against miR-27b-5p or miR-324-5p or a negative control inhibitor. Neurons were cultured in Neurobasal media with B27 supplement. Total RNA was extracted from DIV8-14 neurons using Sepasol RNA I Super G according to the manufacturer's instructions (Nacalai Tesque) and 500 μl of Sepasol was added per well. Five nanograms of total RNA was reverse transcribed using the Taqman miRNA RT kit and RT primers specific for Y1 scRNA (control), miR-196a-5p, miR-27b-5p, miR-324-5p from the Taqman miRNA assay (Applied Biosystems). miRNA-specific qPCR probes and Taqman 2× PCR master mix (Applied Biosystems) were used to detect levels of mature miRNAs. qPCR reactions were set up in triplicates according to the manufacturer's protocol and reactions were carried out on the Bio-Rad CFX-96 machine (Bio-Rad). miRNA levels were normalized to the Y1 scRNA.

### Statistics

Average data are represented as means ± SEM. Statistical significance was determined using two-tailed unpaired *t*-tests on data sets obtained from cell populations. One-Way ANOVA was used when simultaneously comparing three or more data sets. In this case, *p*-values were derived from a *post-hoc* Bonferroni test. Two-Way ANOVA was used to assess difference between treatment conditions (± LRRTM2) and difference between independent neuronal preparations (Figure 3).

### Conflict of interest statement

The authors declare that the research was conducted in the absence of any commercial or financial relationships that could be construed as a potential conflict of interest.

## Supplementary material

Supplemental Figure 1 **Overview of experimental setup of co-culture assay and segmentation strategy. (A)** Experimental timeline. **(B)** The script isolates presynaptic clusters (red dots) formed upon contact of a GFP-transfected axon (green) with HEK293T cells expressing a synaptogenic cue (gray). Endogenous presynapses (black dots) are excluded from the analysis.

Supplemental Figure 2 **Impact of intensity thresholding on segmentation outcomes. (A)** Raw confocal images of GFP-expressing neurons cultured with HEK293T cells transfected with mCherry and the synaptogenic cue LRRTM2 and immunostained against synaptobrevin. Output from two different GFP thresholds. Arrow, additional neuronal process isolated. ^*^, background signal isolated. **(B)** Raw confocal images (identical to Figure 3A bottom row) of GFP-expressing neurons cultured with HEK293T cells transfected with mCherry and LRRTM2 and immunostained against synaptobrevin. Output from two different SV-clusters thresholds. Arrow, additional SV cluster isolated.

Supplemental Figure 3 **Overexpression and knockdown of miRNAs in primary neurons using lentiviruses and miRNA inhibitors, respectively. (A)** Total RNA was extracted from DIV8 hippocampal neurons transduced with lentiviruses expressing a control plasmid (CT), miR-196a, miR-27b, or miR-324. miRNA levels were measured by RT-qPCR using miRNA-specific Taqman probes and internally normalized to the Y1 scRNA. *n* = 2–3. ^*^*p* < 0.05, *t*-test. Expression of all three miRNAs was increased by addition of lentiviruses, but the extent of increase varied. **(B)** Luciferase activity was measured in DIV5-6 cortical neurons transfected with miR-196a-5p or miR-196a-3p luciferase sensors and transduced with control lentivirus (CT) or lentivirus overexpressing miR-196a. Luciferase activity is normalized to CT. *n* = 2. ^***^*p* < 0.0001, ANOVA. **(C)** Total RNA was extracted from DIV10-14 hippocampal or cortical neurons transfected with a negative control miRNA inhibitor (CT KD) or inhibitors against miR-27b-5p or miR-324. miRNA levels were measured by RT-qPCR using miRNA-specific Taqman probes and internally normalized to the Y1 scRNA. Level of mature miRNA was set to 1 for CT KD neurons. *n* = 2. ^**^*p* < 0.005, ANOVA.

Supplemental Figure 4 **Overexpression of miR-196a or miR-27b has no significant effect on the number or size of synaptobrevin or homer puncta in mature hippocampal neurons**. Hippocampal neurons were transduced with viruses expressing the empty vector (CT), miR-196a, or miR-27b, and immunostained (DIV21) against pre- and post-synaptic markers. Effect of overexpression of miR-196a and miR-27b on SV protein synaptobrevin **(A,B)** or postsynaptic protein homer **(C,D)**. *n* = 3. *p* > 0.05, ANOVA.

Supplemental Data 1 **.m files available for the following scripts - SVclusters_detection.m, SVclusters_detection_thresholds.m, and synapse_detection.m**.
