# Supplementary figures and images for: High-content imaging of presynaptic assembly
Source: Front Cell Neurosci. 2014 Mar 3;8:66. doi: 10.3389/fncel.2014.00066 (PMC3939450; doi:10.3389/fncel.2014.00066)

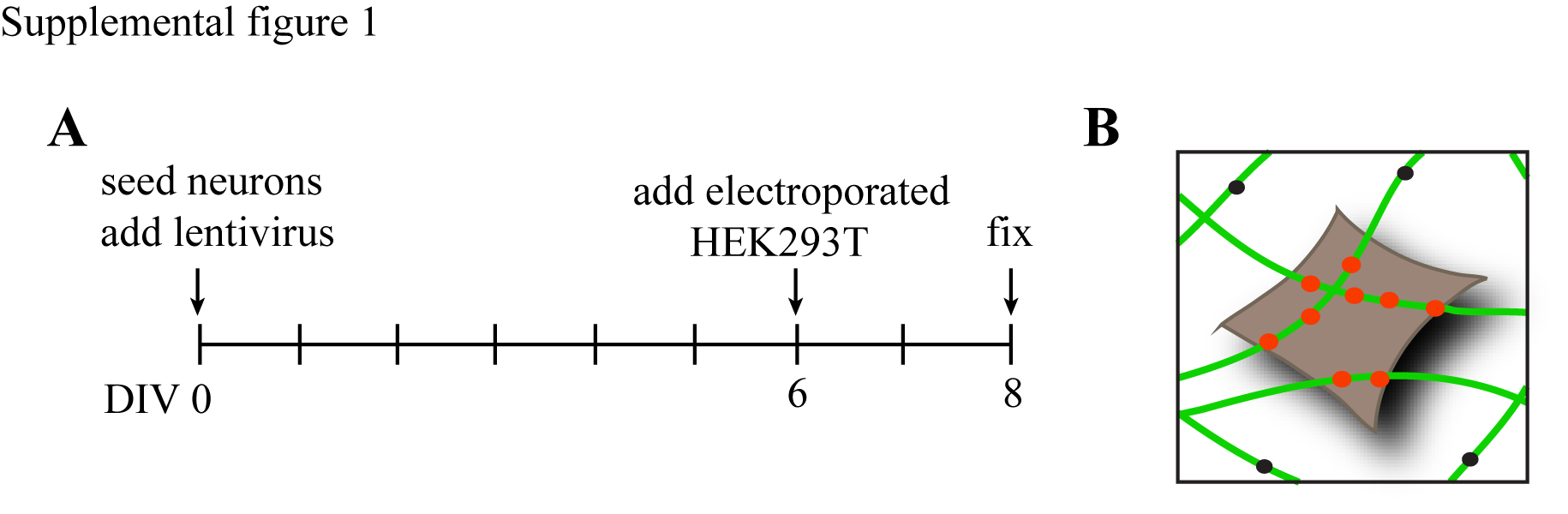

Supplement: Supplemental Figure 1 — Overview of experimental setup of co-culture assay and segmentation strategy. (A) Experimental timeline. (B) The script isolates presynaptic clusters (red dots) formed upon contact of a GFP-transfected axon (green) with HEK293T cells expressing a synaptogenic cue (gray). Endogenous presynapses (black dots) are excluded from the analysis. [file Presentation1.ZIP › 78097_Fivaz_Suppl_Figure_1.TIF]

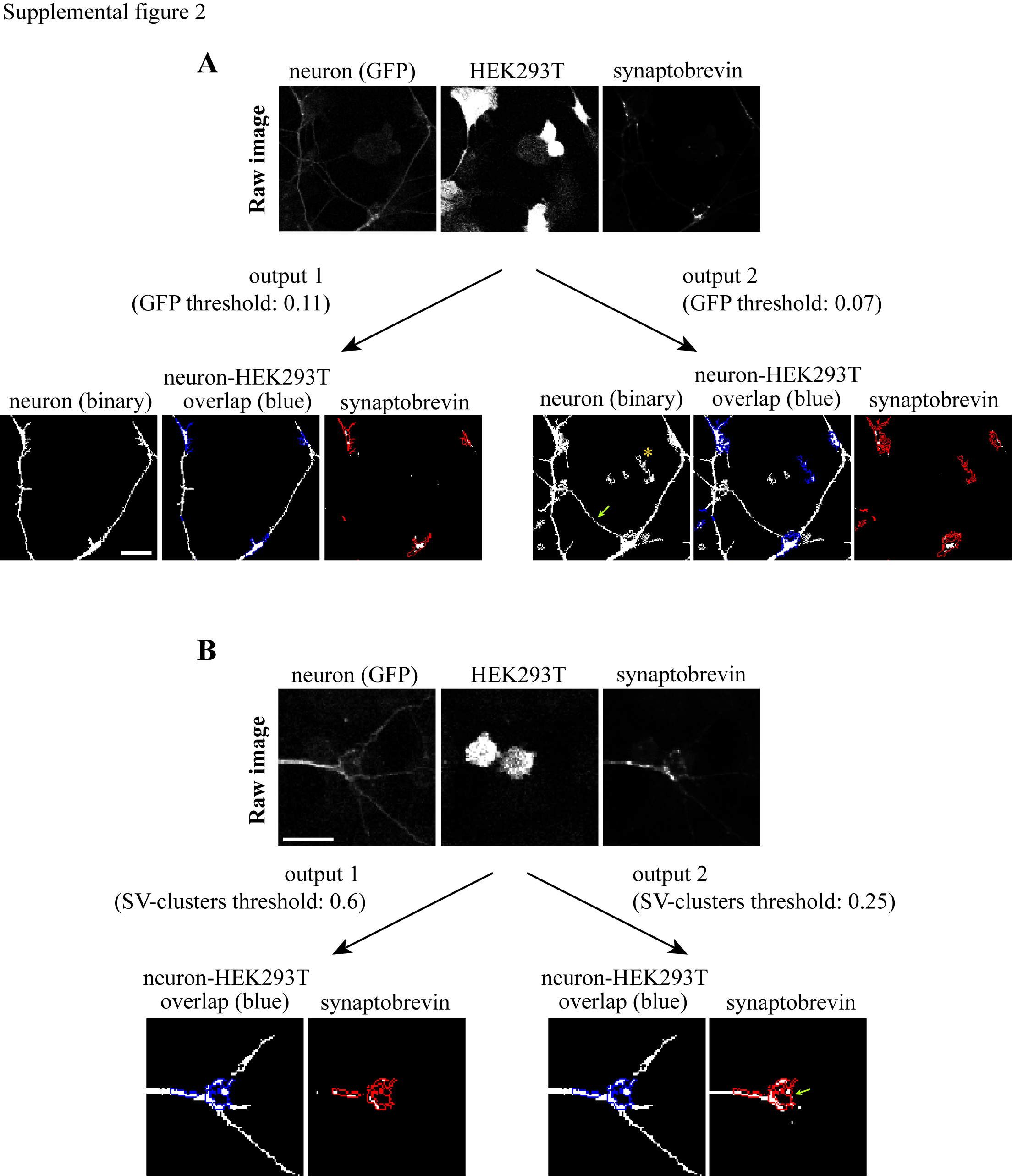

Supplement: Supplemental Figure 1 — Overview of experimental setup of co-culture assay and segmentation strategy. (A) Experimental timeline. (B) The script isolates presynaptic clusters (red dots) formed upon contact of a GFP-transfected axon (green) with HEK293T cells expressing a synaptogenic cue (gray). Endogenous presynapses (black dots) are excluded from the analysis. [file Presentation1.ZIP › 78097_Fivaz_Suppl_Figure_2.TIF]

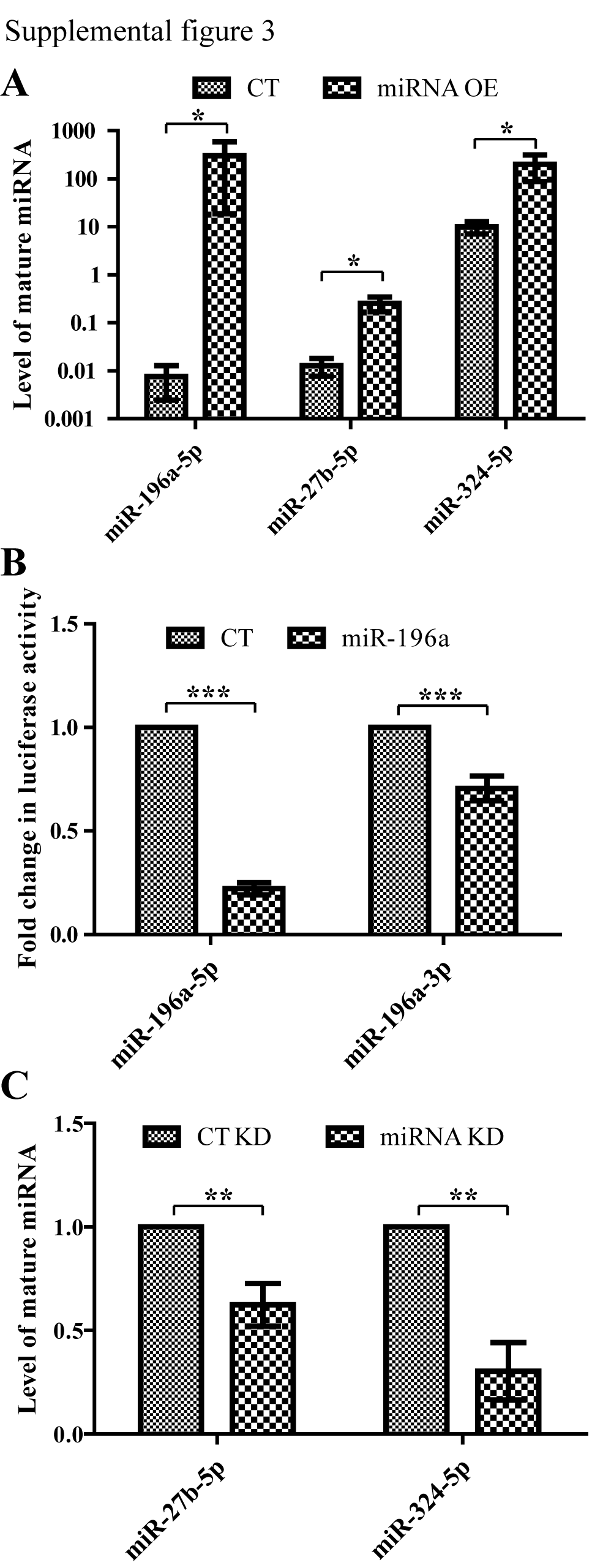

Supplement: Supplemental Figure 1 — Overview of experimental setup of co-culture assay and segmentation strategy. (A) Experimental timeline. (B) The script isolates presynaptic clusters (red dots) formed upon contact of a GFP-transfected axon (green) with HEK293T cells expressing a synaptogenic cue (gray). Endogenous presynapses (black dots) are excluded from the analysis. [file Presentation1.ZIP › 78097_Fivaz_Suppl_Figure_3.TIF]

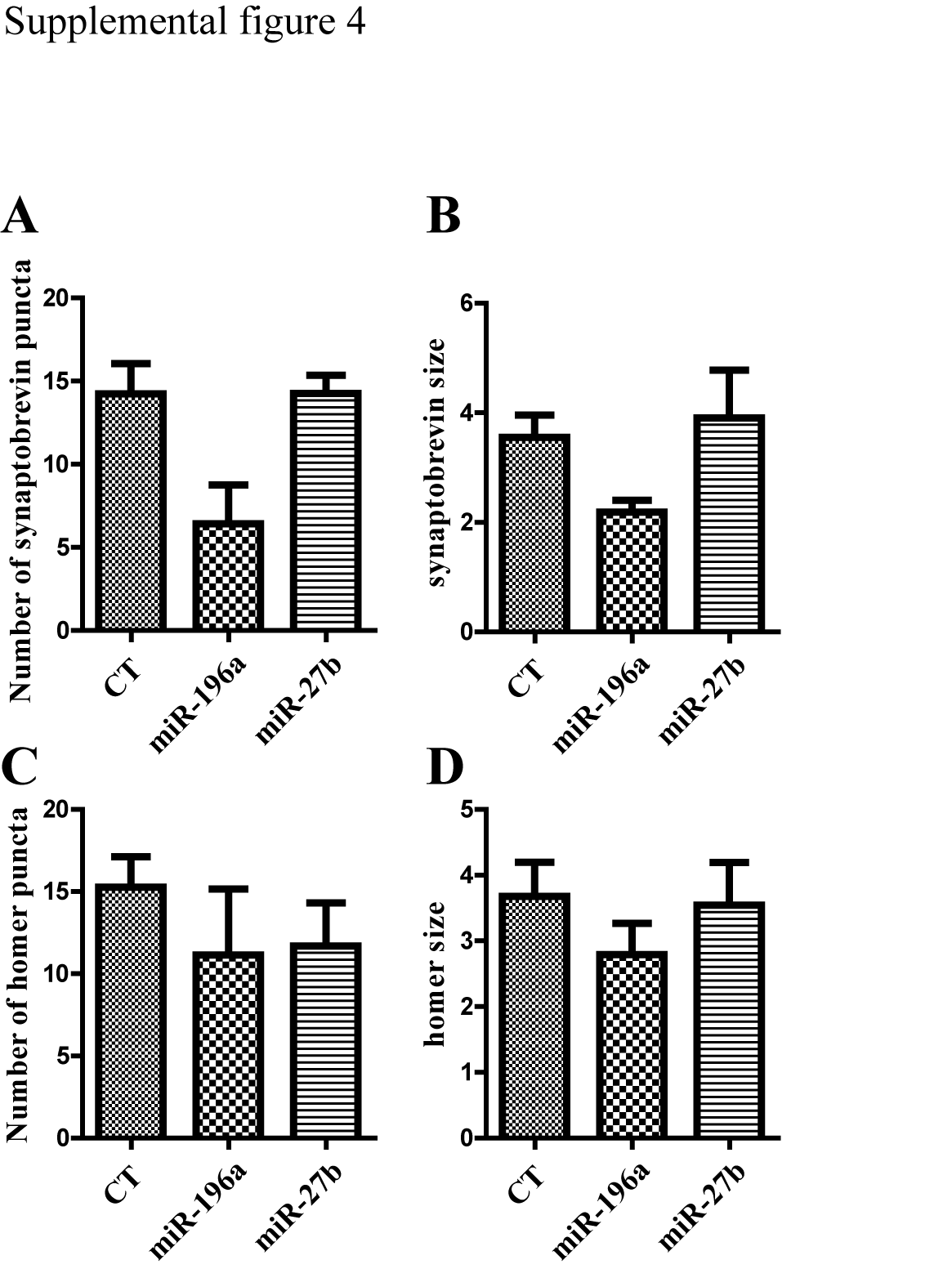

Supplement: Supplemental Figure 1 — Overview of experimental setup of co-culture assay and segmentation strategy. (A) Experimental timeline. (B) The script isolates presynaptic clusters (red dots) formed upon contact of a GFP-transfected axon (green) with HEK293T cells expressing a synaptogenic cue (gray). Endogenous presynapses (black dots) are excluded from the analysis. [file Presentation1.ZIP › 78097_Fivaz_Suppl_Figure_4.TIF]
