# Supplementary material for: High-content imaging of presynaptic assembly
Source: Front Cell Neurosci. 2014 Mar 3;8:66. doi: 10.3389/fncel.2014.00066 (PMC3939450; doi:10.3389/fncel.2014.00066)
Supplement: Supplemental Data 1 — .m files available for the following scripts - SVclusters_detection.m, SVclusters_detection_thresholds.m, and synapse_detection.m. [file DataSheet1.DOCX]

**Supplemental Data 1. Matlab scripts**

**SVclusters_detection.m**

% This script identifies SV clusters in neuron-HEK293T overlap regions.

clear all, close all

% reads Zeiss .lsm file with three color input

i = imread('g lrr5.lsm'); % input file name

i1 = i(:,:,1); % 488nm, gfp-expressing neurons

i1_adj = imadjust(i1);

i2 = i(:,:,2); % 568nm, mcherry-expressing HEK293T cells

i2_adj = imadjust(i2);

i3 = i(:,:,3); % 633nm, synaptobrevin antibody (SV)

i3_adj = imadjust(i3);

figure, imshow(i1_adj), title('gfp-expressing neurons');

figure, imshow(i2_adj), title('mcherry-expressing HEK293T cells');

figure, imshow(i3_adj), title('synaptobrevin (SV)');

%%% set thresholds %%%

i1_threshold_level = 0.11; % threshold for gfp-expressing neurons

i2_threshold_level = 0.4; % threshold for mcherry-expressing HEK293T cells

i3_threshold_level = 0.6; % threshold for SV

detect_size_293 = 5; % threshold for minimum size of mcherry-expressing HEK293T cell

detect_size_neuron = 20; % threshold for minimum size of gfp-expressing neuron

detect_size_SV = 2; % threshold for minimum size of SV cluster

cell_body_size = 7; % value to isolate and remove cell bodies

Overlap_area_filter = 20; % threshold for minimum size of neuron-293T overlap region

%%% binary image for HEK293T cells %%%

bw = im2bw(i2, i2_threshold_level);

%figure, imshow(bw), title('HEK293T cells (binary)');

bw2 = imfill(bw,'holes');

bw3 = bwareaopen(bw2, detect_size_293);

bw3_perim = bwperim(bw3);

se = strel('disk', 3); % dilate HEK293T cells

bw4 = imdilate(bw3, se);

bw5 = imfill(bw4,'holes');

bw5_perim = bwperim(bw5);

overlay2 = imoverlay(i2, bw3_perim, [1 0 0]);

overlay3 = imoverlay(i2, bw5_perim, [1 0 0]);

figure, imshow(overlay2), title('HEK293T cells');

figure, imshow(overlay3), title('HEK293T cells (dilated)');

%%% binary image for gfp-expressing neurons %%%

bw_n = im2bw(i1, i1_threshold_level);

bw_n = bwareaopen(bw_n, detect_size_neuron); % remove small objects

%figure, imshow(bw_n);

% elimination of cell bodies by morphological opening

se_cb = strel('disk', cell_body_size);

bw_n_cb = imerode(bw_n, se_cb);

bw_n_cb = imdilate(bw_n_cb, se_cb);

bw_n = bw_n - bw_n_cb;

figure, imshow(bw_n);

%%% compute neuron-HEK293T overlap regions %%%

overlay_neuron_293 = imoverlay(bw_n, bw5_perim, [1 0 0]);

figure, imshow(overlay_neuron_293), title('overlay of neurons and HEK293T cells');

ax_bw_293 = bw_n & bw5;

ax_bw_293 = bwareaopen(ax_bw_293, Overlap_area_filter);

ax_bw_293_perim = bwperim(ax_bw_293);

overlay_ax_bw_293= imoverlay(bw_n, ax_bw_293_perim, [0 0 1]);

figure, imshow(overlay_ax_bw_293), title('neuron-HEK293T overlap regions');

% label neuron-HEK293T overlap regions

[L_ax_bw_293, num_ax_bw_293] = bwlabel(ax_bw_293);

stats_ax_bw_293 = regionprops(L_ax_bw_293, 'PixelIdxList', 'Area');

figure, vislabels(L_ax_bw_293), title('numbering of neuron-HEK293T overlap regions');

RGB_ov = label2rgb(L_ax_bw_293, 'cool');

figure, imshow(RGB_ov), title('labeled overlap regions')

%%% binary image for SV clusters

bw_sc = im2bw(i3, i3_threshold_level);

bw_sc = bwareaopen(bw_sc, detect_size_SV);

overlay4 = imoverlay(bw_sc, ax_bw_293_perim, [1 0 0]);

figure, imshow(overlay4), title('overlay of SV clusters and neuron-HEK293T overlap regions');

bw_sc_c = imcomplement(bw_sc);

%%% displaying SV clusters on Neuron-overlap map (using Transparency)

alpha_data = bw_sc;

figure, imshow(RGB_ov);

hold on

h = imshow(bw_sc_c);

hold off

set(h, 'AlphaData', alpha_data);

% list of SV parameters

count = 0;

axon_number = []; % labeled neuron-HEK293T overlap region

av_int= []; % mean SV intensity

cluster_size = []; % size of SV clusters within neuron-HEK293T overlap region

axon_area = []; % area of neuron-HEK293T overlap region

cluster_density = []; % fraction of area of neuron-HEK293T overlap region occupied by SV clusters

% measures SV parameters for each neuron-HEK293T overlap region

for j=1:num_ax_bw_293

count = count + 1;

axon_number(count) = j;

av_int(count)= mean(i3(stats_ax_bw_293(j).PixelIdxList));

cluster_size(count) = sum(bw_sc(stats_ax_bw_293(j).PixelIdxList));

axon_area(count) = sum(ax_bw_293(stats_ax_bw_293(j).PixelIdxList));

cluster_density(count) = (cluster_size(count)*100)./axon_area(count);

end

% exports results to excel file

output=[axon_number', av_int', cluster_size', axon_area', cluster_density'];

xlswrite('SV-clusters',output);

**SVclusters_detection_thresholds.m**

% This script identifies SV clusters in neuron-HEK293T overlap regions.

clear all, close all

% reads Zeiss .lsm file with three color input

i = imread('g lrr5.lsm'); % input file name

i1 = i(:,:,1); % 488nm, gfp-expressing neurons

i1_adj = imadjust(i1);

i2 = i(:,:,2); % 568nm, mcherry-expressing HEK293T cells

i2_adj = imadjust(i2);

i3 = i(:,:,3); % 633nm, synaptobrevin antibody (SV)

i3_adj = imadjust(i3);

figure, imshow(i1_adj), title('gfp-expressing neurons');

figure, imshow(i2_adj), title('mcherry-expressing HEK293T cells');

figure, imshow(i3_adj), title('synaptobrevin (SV)');

%%% set gating parameters %%%

detect_size_293 = 5; % threshold for minimum size of mcherry-expressing HEK293T cell

detect_size_neuron = 20; % threshold for minimum size of gfp-expressing neuron

detect_size_SV = 2; % threshold for minimum size of SV cluster

cell_body_size = 7; % value to isolate and remove cell bodies

Overlap_area_filter = 20; % threshold for minimum size of neuron-293T overlap region

%%% binary image for HEK293T cells %%%

[level, bw] = thresh_tool(i2); % GUI thresholding tool

%figure, imshow(bw), title('HEK293T cells (binary)');

bw2 = imfill(bw,'holes');

bw3 = bwareaopen(bw2, detect_size_293);

bw3_perim = bwperim(bw3);

se = strel('disk', 3); % dilate HEK293T cells

bw4 = imdilate(bw3, se);

bw5 = imfill(bw4,'holes');

bw5_perim = bwperim(bw5);

overlay2 = imoverlay(i2, bw3_perim, [1 0 0]);

overlay3 = imoverlay(i2, bw5_perim, [1 0 0]);

figure, imshow(overlay2), title('HEK293T cells');

figure, imshow(overlay3), title('HEK293T cells (dilated)');

%%% binary image for gfp-expressing neurons %%%

[level, bw_n] = thresh_tool(i1);

bw_n = bwareaopen(bw_n, detect_size_neuron); % remove small objects

figure, imshow(bw_n);

% elimination of cell bodies by morphological opening

se_cb = strel('disk', cell_body_size);

bw_n_cb = imerode(bw_n, se_cb);

bw_n_cb = imdilate(bw_n_cb, se_cb);

bw_n = bw_n - bw_n_cb;

figure, imshow(bw_n);

%%% compute neuron-HEK293T overlap regions %%%

overlay_neuron_293 = imoverlay(bw_n, bw5_perim, [1 0 0]);

figure, imshow(overlay_neuron_293), title('overlay of neurons and HEK293T cells');

ax_bw_293 = bw_n & bw5;

ax_bw_293 = bwareaopen(ax_bw_293, Overlap_area_filter);

ax_bw_293_perim = bwperim(ax_bw_293);

overlay_ax_bw_293= imoverlay(bw_n, ax_bw_293_perim, [0 0 1]);

figure, imshow(overlay_ax_bw_293), title('neuron-HEK293T overlap regions');

% label neuron-HEK293T overlap regions

[L_ax_bw_293, num_ax_bw_293] = bwlabel(ax_bw_293);

stats_ax_bw_293 = regionprops(L_ax_bw_293, 'PixelIdxList', 'Area');

figure, vislabels(L_ax_bw_293), title('numbering of neuron-HEK293T overlap regions');

RGB_ov = label2rgb(L_ax_bw_293, 'cool');

figure, imshow(RGB_ov), title('labeled overlap regions')

%%% binary image for SV clusters

[level, bw_sc] = thresh_tool(i3);

bw_sc = bwareaopen(bw_sc, detect_size_SV);

overlay4 = imoverlay(bw_sc, ax_bw_293_perim, [1 0 0]);

figure, imshow(overlay4), title('overlay of SV clusters and neuron-HEK293T overlap regions');

bw_sc_c = imcomplement(bw_sc);

%%% displaying SV clusters on Neuron-overlap map (using Transparency)

alpha_data = bw_sc;

figure, imshow(RGB_ov);

hold on

h = imshow(bw_sc_c);

hold off

set(h, 'AlphaData', alpha_data);

% list of SV parameters

count = 0;

axon_number = []; % labeled neuron-HEK293T overlap region

av_int= []; % mean SV intensity

cluster_size = []; % size of SV clusters within neuron-HEK293T overlap region

axon_area = []; % area of neuron-HEK293T overlap region

cluster_density = []; % fraction of area of neuron-HEK293T overlap region occupied by SV clusters

% measures SV parameters for each neuron-HEK293T overlap region

for j=1:num_ax_bw_293

count = count + 1;

axon_number(count) = j;

av_int(count)= mean(i3(stats_ax_bw_293(j).PixelIdxList));

cluster_size(count) = sum(bw_sc(stats_ax_bw_293(j).PixelIdxList));

axon_area(count) = sum(ax_bw_293(stats_ax_bw_293(j).PixelIdxList));

cluster_density(count) = (cluster_size(count)*100)./axon_area(count);

end

% exports results to excel file

output=[axon_number', av_int', cluster_size', axon_area', cluster_density'];

xlswrite('SV-clusters',output);

**synapse_detection.m**

% This script identifies overlap regions between pre- and post-synaptic markers within neuronal process

clear all, close all;

% set thresholds

level_gfp = 0.2; % threshold for gfp-expressing neuron

level_gfp_skeleton = 0.2; % threshold for determining dendrite length

gfp_size = 1000; % threshold for minimum size of gfp-expressing neuron

se_gfp = 1; % extent of dilation of gfp-expressing neuron

level_homer = 0.25; % threshold for post-synaptic marker

level_VAMP2 = 0.4; % threshold for pre-synaptic marker

homer_min = 1; % threshold for minimum size for post-synaptic marker

homer_max = 100; % threshold for maximum size for post-synaptic marker

vamp_min = 1; % threshold for minimum size for pre-synaptic marker

vamp_max = 100; % threshold maximum size for pre-synaptic marker

% read 16-bit tif file with three color input

i = imread('.tif');

gfp = i(:,:,1); % 488nm, gfp-expressing neurons

VAMP2 = i(:,:,2); % 568nm, vamp2 antibody

homer = i(:,:,3); % 633nm, homer antibody

% expand signal range

max_gfp = double(max(max(gfp)))/65535;

max_homer = double(max(max(homer)))/65535;

max_VAMP2 = double(max(max(VAMP2)))/65535;

gfp_adj = imadjust(gfp, [0 max_gfp*0.75], []);

homer_adj = imadjust(homer, [0 max_homer*0.75], []);

VAMP2_adj = imadjust(VAMP2, [0 max_VAMP2*0.75], []);

figure, imshow(gfp_adj), title('neuron');

figure, imshow(homer_adj), title('homer');

figure, imshow(VAMP2_adj), title('vamp2');

% binary image clean-up (neuronal process)

bw = im2bw(gfp, level_gfp);

figure, imshow(bw);

bw2 = imfill(bw,'holes');

bw3 = bwareaopen(bw, gfp_size);

figure, imshow(bw3), title('neuron (binary)');

se = strel('disk', se_gfp);

bw4 = imdilate(bw3, se);

figure, imshow(bw4), title('dilated neuron (binary)');

bw4_perim = bwperim(bw4);

overlay_ho = imoverlay(homer_adj, bw4_perim, [1 0 0]);

overlay_va = imoverlay(VAMP2_adj, bw4_perim, [1 0 0]);

figure, imshow(overlay_ho), title('overlay of homer and neuron');

figure, imshow(overlay_va), title('overlay of VAMP2 and neuron');

% remove of homer and VAMP2 puncta that are not within gfp-positive neuron

bw4_i = uint16(bw4 .* 65535);

homer_ov = min(bw4_i, homer);

homer_ov = uint16(round(homer_ov));

VAMP2_ov = min(bw4_i, VAMP2);

VAMP2_ov = uint16(round(VAMP2_ov));

% binary image clean-up (homer)

bw_homer = im2bw(homer_ov, level_homer);

bw_homer = imfill(bw_homer,'holes');

bw_homer = bwareaopen(bw_homer,homer_min);

bw_plus = bwareaopen(bw_homer,homer_max);

bw_homer = bw_homer - bw_plus;

overlay_ho1 = imoverlay(bw_homer, bw4_perim, [1 0 0]);

figure, imshow(overlay_ho1), title('overlay of homer(binary) and neuron');

[L5, num5] = bwlabel(bw_homer);

stats5 = regionprops(L5, 'PixelIdxList', 'Area');

figure, vislabels(L5), title('numbering of homer');

for k=1:num5

homer_int(k)= mean(homer_ov(stats5(k).PixelIdxList));

homer_size(k) = sum(bw_homer(stats5(k).PixelIdxList));

end

% binary image clean-up (VAMP2)

bw_VAMP2 = im2bw(VAMP2_ov, level_VAMP2);

bw_VAMP2 = imfill(bw_VAMP2,'holes');

bw_VAMP2 = bwareaopen(bw_VAMP2,vamp_min);

bw_plus = bwareaopen(bw_VAMP2,vamp_max);

bw_VAMP2 = bw_VAMP2 - bw_plus;

overlay_va1 = imoverlay(bw_VAMP2, bw4_perim, [1 0 0]);

figure, imshow(overlay_va1);

title('VAMP2 binary + dil binary im');

[L4, num4] = bwlabel(bw_VAMP2);

stats4 = regionprops(L4, 'PixelIdxList', 'Area');

figure, vislabels(L4), title('numbering of VAMP2');

for l=1:num4

%vamp_int(l)= mean(VAMP2_ov(stats4(l).PixelIdxList));

vamp_size(l) = sum(bw_VAMP2(stats4(l).PixelIdxList));

end

% identify overlap regions between homer and VAMP2

synapse_count = bw_homer & bw_VAMP2;

figure, imshow(synapse_count), title('homer-VAMP2 overlap');

overlay_syn = imoverlay(synapse_count, bw4_perim, [1 0 0]);

figure, imshow(overlay_syn), title('overlay of homer-VAMP2 overlap and neuron');

[L, num] = bwlabel(synapse_count);

stats = regionprops(L, 'PixelIdxList', 'Area');

figure, vislabels(L), title('labeling homer-VAMP2 overlap');

% determine neuronal surface area

[L2, num2] = bwlabel(bw3);

stats2 = regionprops(L2, 'PixelIdxList', 'Area');

figure, vislabels(L2), title('neuronal surface area');

for j=1:num2

gfp_area(j) = sum(bw4(stats2(j).PixelIdxList));

end

tot_area = sum(gfp_area(:));

synapse_number = num; % number of homer-VAMP2 overlap regions

synapse_density = num*10000/tot_area; % number of homer-VAMP2 overlap regions normalized to dendritic surface area

vamp_number = num4; % number of VAMP2 puncta

vamp_density = num4*10000/tot_area; % number of VAMP2 puncta normalized to dendritic surface area

homer_number = num5; % number of homer puncta

homer_density = num5*10000/tot_area; % number of homer puncta normalized to dendritic surface area

% determine neuronal process (dendrite) length

bw5 = im2bw(gfp, level_gfp_skeleton);

bw5 = bwareaopen(bw5, gfp_size);

bw5 = medfilt2(bw5, [3 3]);

bw5_tk = bwmorph(bw5, 'thicken', 2);

bw5_tk = medfilt2(bw5_tk, [10 10]);

bw5_th = bwmorph(bw5_tk, 'thin', Inf);

bw8 = bw5_th;

[L3, num3] = bwlabel(bw8);

stats3 = regionprops(L3, 'PixelIdxList');

figure, vislabels(L3), title ('dendrite');

for i=1:num3

dendr_length(i) = sum(bw8(stats3(i).PixelIdxList));

end

pixelDim=0.16; % Value depend on microscope used

dendrite_length = sum(dendr_length) * pixelDim;

synapse_density2 = num*10/dendrite_length; % number of homer-VAMP2 overlap regions per 10um dendrite length

vamp_density2 = num4*10/dendrite_length; % number of VAMP2 puncta per 10um dendrite length

homer_density2 = num5*10/dendrite_length; % number of homer puncta per 10um dendrite length

% exports results to excel file

data = [vamp_number, homer_number, synapse_number, dendrite_length, vamp_density2, homer_density2, synapse_density2, tot_area, vamp_density, homer_density, synapse_density, sum(vamp_size), mean(vamp_size), sum(homer_size), mean(homer_size)];

xlswrite('synapse',data);
